# Facile fabrication of superparamagnetic graphene/polyaniline/Fe_3_O_4_ nanocomposites for fast magnetic separation and efficient removal of dye

**DOI:** 10.1038/s41598-017-05755-6

**Published:** 2017-07-13

**Authors:** Bin Mu, Jie Tang, Long Zhang, Aiqin Wang

**Affiliations:** 1 State Key Laboratory of Solid Lubrication, Center of Eco-materials and Green Chemistry, Lanzhou Institute of Chemical Physics, Chinese Academy of Sciences, Lanzhou, 730000 China; 2 University of Chinese Academy of Sciences, Beijing, 100049 China; 3 School of Material Science and Engineering, Lanzhou University of Technology, Lanzhou, 730050 China

## Abstract

Using graphene as adsorbent for removal of pollutants from polluted water is commonly recognized to be costly because the graphene is usually produced by a very complex process. Herein, a simple and eco-friendly method was employed to fabricate efficient superparamagnetic graphene/polyaniline/Fe_3_O_4_ nanocomposites for removal of dyes. The exfoliation of graphite as nanosheets and the functionalization of nanosheets with polyaniline and Fe_3_O_4_ nanoparticles were simultaneously achieved *via* a one-pot reaction process combining the intercalation polymerization of aniline and the co-precipitation of the residual Fe^3+^ and the generated Fe^2+^. The obtained graphene/polyaniline/Fe_3_O_4_ nanocomposites exhibited excellent adsorption performance for Congo red, even in the presence of Brilliant green. The adsorption kinetics and adsorption isotherms were well fitted with pseudo second-order kinetic model and Langmuir isotherm model, respectively. In a word, this method is simple and industrially feasible, which provides a new approach to fabricate highly efficient graphene-based adsorbents on large scale for removal of dyes. In addition, it also can be used to exfoliate other two-dimensional materials, such as boron nitride, carbon nitride and MoS_2_ for a range of possible applications.

## Introduction

Graphene is a two-dimensional sheet material with atomic thickness composed of the honeycomb rings formed by sp^2^-bonded carbon atoms^1, 2^. The large theoretical specific surface area of graphene (about 2630 m^2^ g^−1^) and the abundant functional groups of graphene oxide (GO) make them potential to be used for removal of heavy metal ions and organic pollutants^3, 4^. Currently, several strategies have been employed to prepare GO, including “Scotch tape” method^5^, chemical vapor deposition^6^, epitaxial growth on substrates^7^, exfoliation from expanded graphite^8^ and Hummers’ method^9^. Among them, chemical exfoliation method has been frequently used for large-scale production of graphene^10^, because the other methods have drawbacks such as high cost, low yield and harsh reaction conditions (i.e., high temperature and vacuum)^11, 12^. However, the conventional chemical exfoliation method is subject to be limited because it needs complex procedures including oxidation, intercalation, exfoliation and/or reduction of graphene derivatives^13^. In addition, graphene and GO are usually difficult to be separated from water by traditional centrifugation and filtration methods due to their small size and good hydrophillicity^14^.

Magnetic separation is a kind of efficient, rapid and inexpensive technique to separate adsorbents from solution. Thus far, magnetic graphene or GO-based adsorbents have been prepared using different approaches for removal of pollutants^14–17^. Jiao *et al*. prepared a magnetic GO-based composite based on the physical affinities between sulfonated GO and Fe_3_O_4_ nanoparticles for efficient removal of organic dyes^18^. Gollavelli *et al*. prepared magnetic graphene composites by microwave radiation of GO and ferrocene precursors for adsorption of heavy metal ions from aqueous solution^19^. Hu *et al*. prepared nitrogen-doped magnetic graphene oxide by three steps for removal of Cu(II) and Cr(VI)^20^. In addition, a one-pot solvothermal method was also employed to produce superparamagnetic graphene-Fe_3_O_4_ nanocomposite for removal of dye^21^. However, multi-step reactions are needed to prepare magnetic graphene-based composites in these methods. The GO should be prepared firstly *via* a long step; and then the magnetic particles and functional groups were introduced by other steps to improve the magnetic property and adsorption capacity. Therefore, it is impractical to produce magnetic graphene-based composites from expensive GO on large scale because of the complex process and high cost^22^.

It is well known that the key problem to exfoliate the graphite layers is to overcome or destroy the strong van der Waals forces between the layers. It has been reported that the intercalation of metal cations^23, 24^ and ring compounds containing *π*-electron^25, 26^ can effectively exfoliate graphite as graphene. Polyaniline (PANI) is a nitrogen-containing conductive polymer. Due to the *π*-electron conjugation structure, PANI can well exfoliate the graphite as graphene sheets by the intercalation polymerization of the protonated aniline (An^+^)^27, 28^. What’s more, the PANI is helpful to enhance the adsorption property of magnetic graphene-based adsorbents due to the introduction of large amounts of amine and imine groups^29, 30^. However, in the previous reports, magnetic graphene/PANI composites were usually fabricated *via* two steps, and graphite needs to be transformed into GO *via* a complex procedure. Liu *et al*. prepared the ternary graphene-polyaniline-Fe_3_O_4_ nanocomposites *via* a two-step method including the chemical oxidative polymerization of aniline using (NH_4_)_2_S_2_O_8_ in the presence of GO and the *in-situ* generation of Fe_3_O_4_ nanoparticles by a co-precipitation method^31^. Li *et al*. fabricated the hierarchical PANI decorated GO/Fe_3_O_4_ composites for the adsorption of ionic dye^32^. However, the GO/Fe_3_O_4_ needs to be firstly synthesized *via* a solvothermal approach. Therefore, it remains a great challenge to develop a mild and simple method to fabricate magnetic graphene/PANI composites for removal of pollutants.

**Figure 1:**
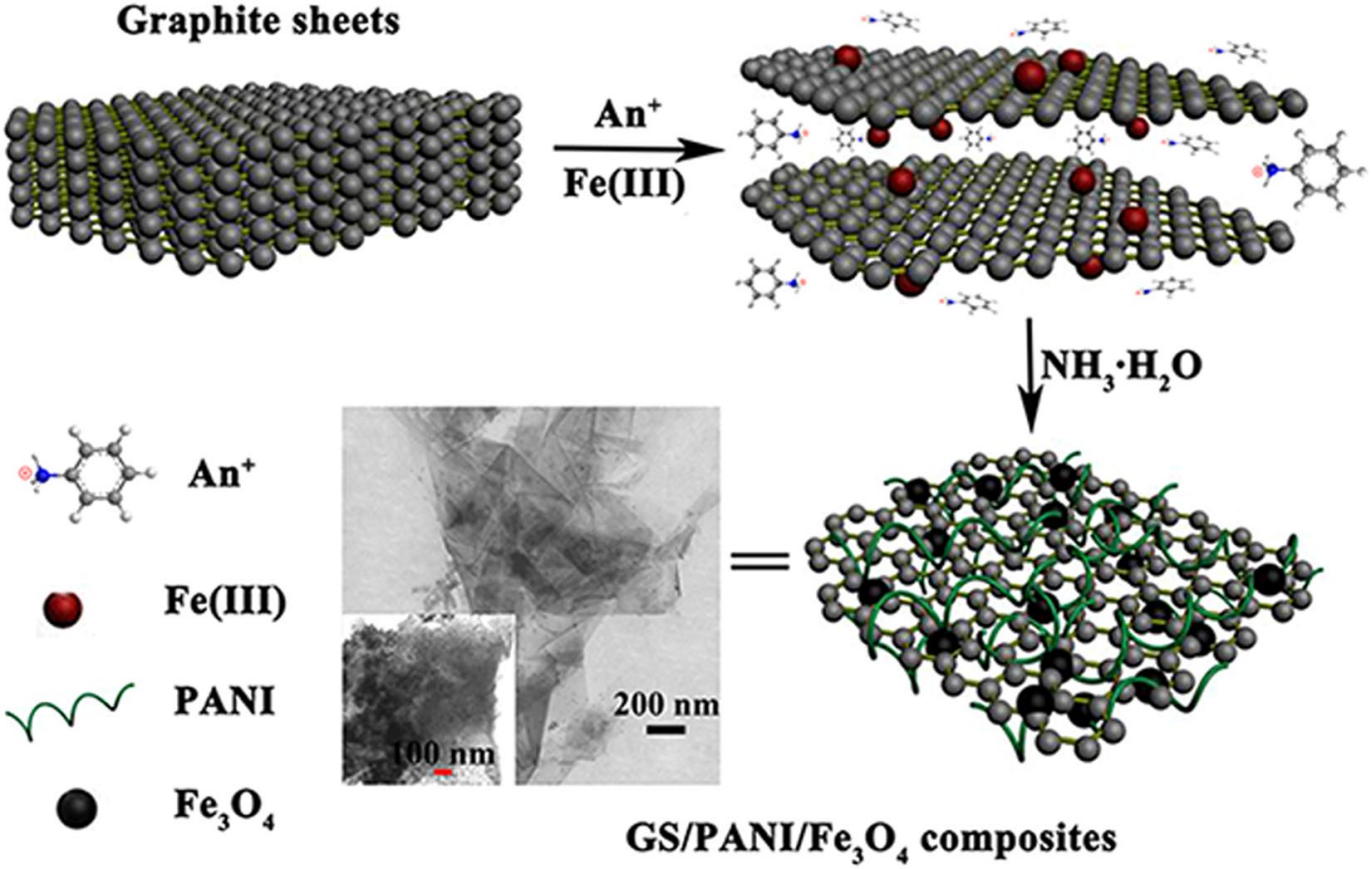
Illustration for the fabrication of superparamagnetic graphene/polyaniline/Fe_3_O_4_ nanocomposites *via in situ* intercalation polymerization and co-precipitation technique.

In our previous study, a facile one-pot method was developed to prepare the superparamagnetic attapulgite/Fe_3_O_4_/PANI nanocomposites^33^ using inexpensive Fe(III) as both of the oxidant for the oxidative polymerization of aniline and the single iron source of Fe_3_O_4_. This strategy inspires us to directly synthesize the magnetic graphene/PANI adsorbents based on the original graphite powder (GP) for removal of pollutants combining the intercalation polymerization of aniline and co-precipitation technique. As illustrated in Fig. 1, the superparamagnetic graphene sheet/polyaniline/Fe_3_O_4_ (GS/PANI/Fe_3_O_4_) nanocomposites were prepared *via* a similar procedure. The batch adsorption tests of the obtained GS/PANI/Fe_3_O_4_ nanocomposites toward dye molecules were carried out to investigate the adsorption properties using Congo red (CR) and Brilliant green (BG) as the model pollutants. In addition, a possible adsorption mechanism was also proposed.

## Results and discussion

### General characterization

**Figure 2:**
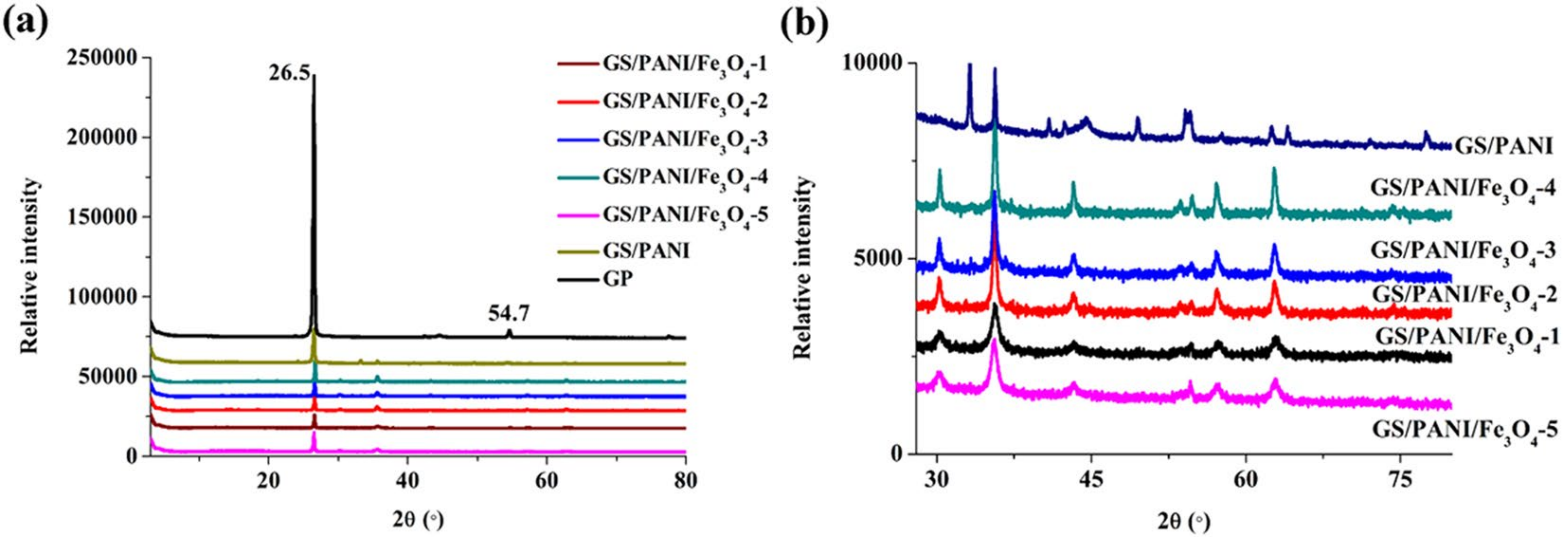
XRD patterns of GP, GS/PANI and GS/PANI/Fe_3_O_4_ nanocomposites.

Figure 2a shows the XRD patterns of natural GP, GS/PANI and GS/PANI/Fe_3_O_4_ nanocomposites. Two sharp characteristic diffraction peaks at 2*θ = *26.5° and 54.7° were observed in the XRD pattern of GP, which were assigned to the (002) and (004) plane of GP, respectively^34^. It was well-known that the diffraction peak of (002) plane of GP became broad or smaller due to the disorganization on *c* axis when it was expanded or intercalated, which could be confirmed by the XRD patterns of GS/PANI and GS/PANI/Fe_3_O_4_ (Fig. 2a). It was clear that this diffraction peak obviously weakened for GS/PANI and GS/PANI/Fe_3_O_4_, suggesting a severe disorganization on *c* axis and the exfoliation of GP into mono or few-layered sheets due to the intercalation polymerization of aniline and the formation of Fe_3_O_4_ nanoparticles^35^. Furthermore, the characteristic diffraction peaks of the HCl-doped PANI crystal at 21.7°, 26.7° and 33.2° were observed in the XRD patterns of GS/PANI and GS/PANI/Fe_3_O_4_ (Fig. S1), indicating the polymer chains were parallel and arranged orderly in a close-packed array^36, 37^.

Several characteristic diffraction peaks at 30.2°, 35.6°, 43.3°, 54.5°, 57.2°, and 62.6°, were observed in the XRD patterns of the GS/PANI/Fe_3_O_4_ nanocomposite in contrast to the XRD pattern of GS/PANI (Fig. 2b), which were ascribed to the (220), (311), (400), (422), (511) and (440) crystalline planes of Fe_3_O_4_, respectively^38, 39^. These peaks were readily identified as the pure cubic phase [space group: *Fd3m* (227)] of Fe_3_O_4_ with the cell constant *a* = 8.389 Å (JCPDS 01–074–1910). Interestingly, the relative intensity of these diffraction peaks firstly increased with increasing the dosage of aniline from 0.6 to 3.6 mL, and then decreased with the further increase in the dosage of aniline. In the preparation process of GS/PANI/Fe_3_O_4_, inexpensive Fe(III) served as oxidant to initiate the chemical oxidative polymerization of aniline, and itself was reduced as Fe(II). The optimal molar ratio of Fe(III) to Fe(II) for formation of Fe_3_O_4_ was 2:1, but the content of Fe(III) in the reaction system decreased, while the content of Fe(II) increased with increasing the dosage of aniline. As a result, the content of Fe_3_O_4_ in the nanocomposites was controlled by the initial molar ratio of aniline to Fe(III). In addition, the average crystallite size of the Fe_3_O_4_ nanoparticles was calculated to be 2.0~5.8 nm according to Debye-Scherrer equation:

1 $$d=\frac{k\times \lambda }{\beta \times \,\cos \,\theta }$$

where *d* is the average crystallite size, *λ* is the wavelength of X-ray, *k* is the shape factor (0.89), *β* is the full width at half maximum and *θ* is the Bragg angle of the diffraction peak.

**Figure 3:**
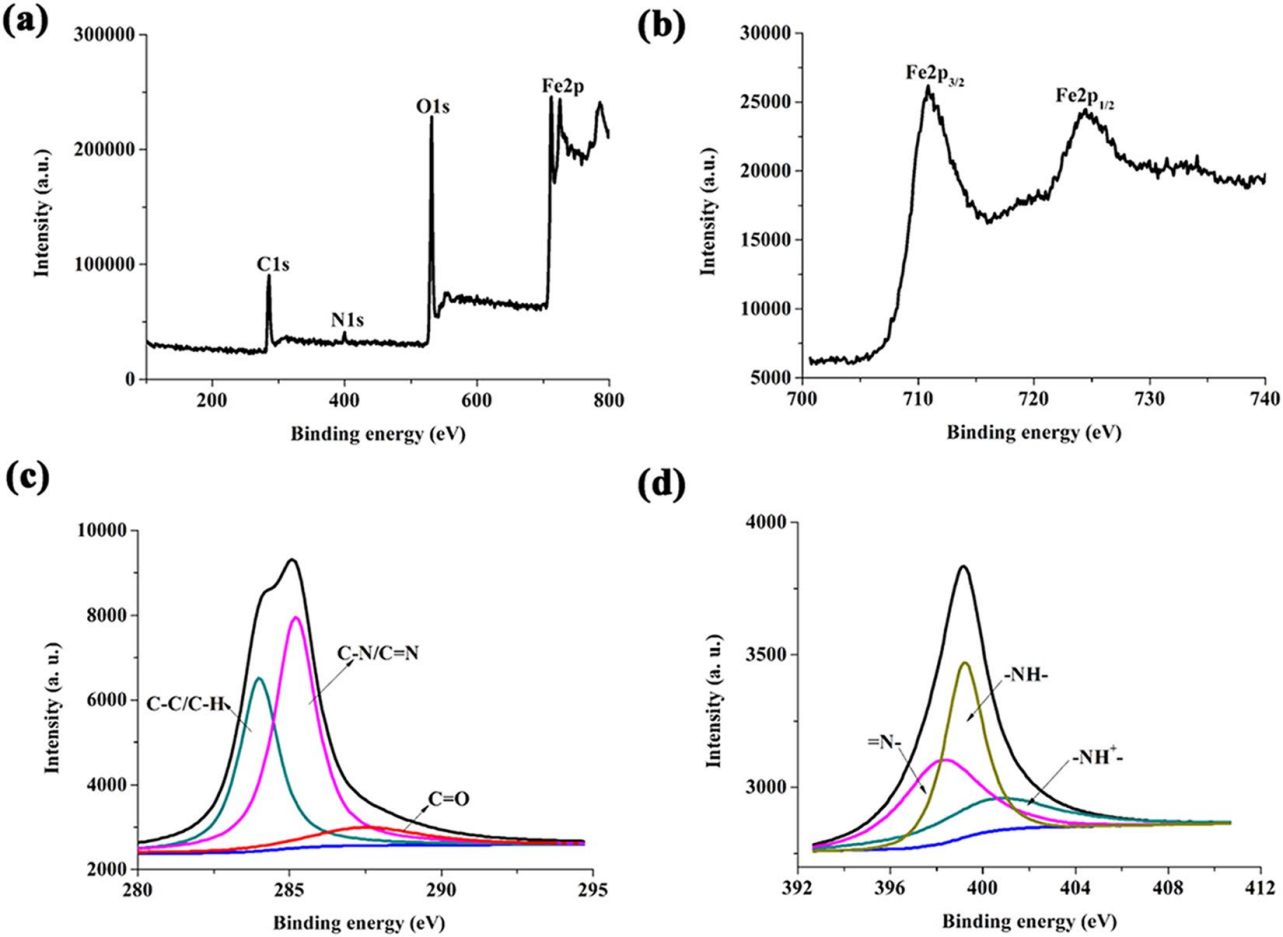
XPS spectra of GS/PANI/Fe_3_O_4_-4 nanocomposite: (**a**) full spectrum, (**b**) Fe2p, (**c**) C1s, and (**d**) N1s.

XPS analysis was performed to evaluate the surface electronic state and the atomic composition of GS/PANI/Fe_3_O_4_−4 nanocomposite. Figure 3a gives the full-scanning XPS spectrum in the range of 0~800 eV and the atomic composition of carbon, nitrogen, oxygen and iron in the GS/PANI/Fe_3_O_4_-4 nanocomposite. The high resolution XPS spectrum of Fe2p for GS/PANI/Fe_3_O_4_-4 nanocomposites displayed the typical doublets of Fe2p_3/2_ and Fe2p_1/2_ core level spectrum of Fe_3_O_4_, corresponding to two broad peaks located at 710.7 and 724.5 eV, respectively (Fig. 3b)^40^. The deconvoluted profile fit of the XPS spectrum for C1s indicated the presence of benzene ring-C (C–C/C–H, 284.0 eV), C–N/C = N (285.2 eV), and C = O of quinoid (287.5 eV)^41^, as shown in Fig. 3c. The N1s core-level spectrum could be deconvoluted into three Gaussian peaks (Fig. 3d), and the peak of major benzenoid-amine component (–NH–) was observed at 399.5 eV along with a small quinoid-imine ( = NH–) peak at 398.9 eV^42^. The shoulder peak at a high binding energy of 400.5 eV was ascribed to positively charged nitrogen (–NH^+^–), suggesting the formation of the protonated nitrogen species^42^.

**Figure 4:**
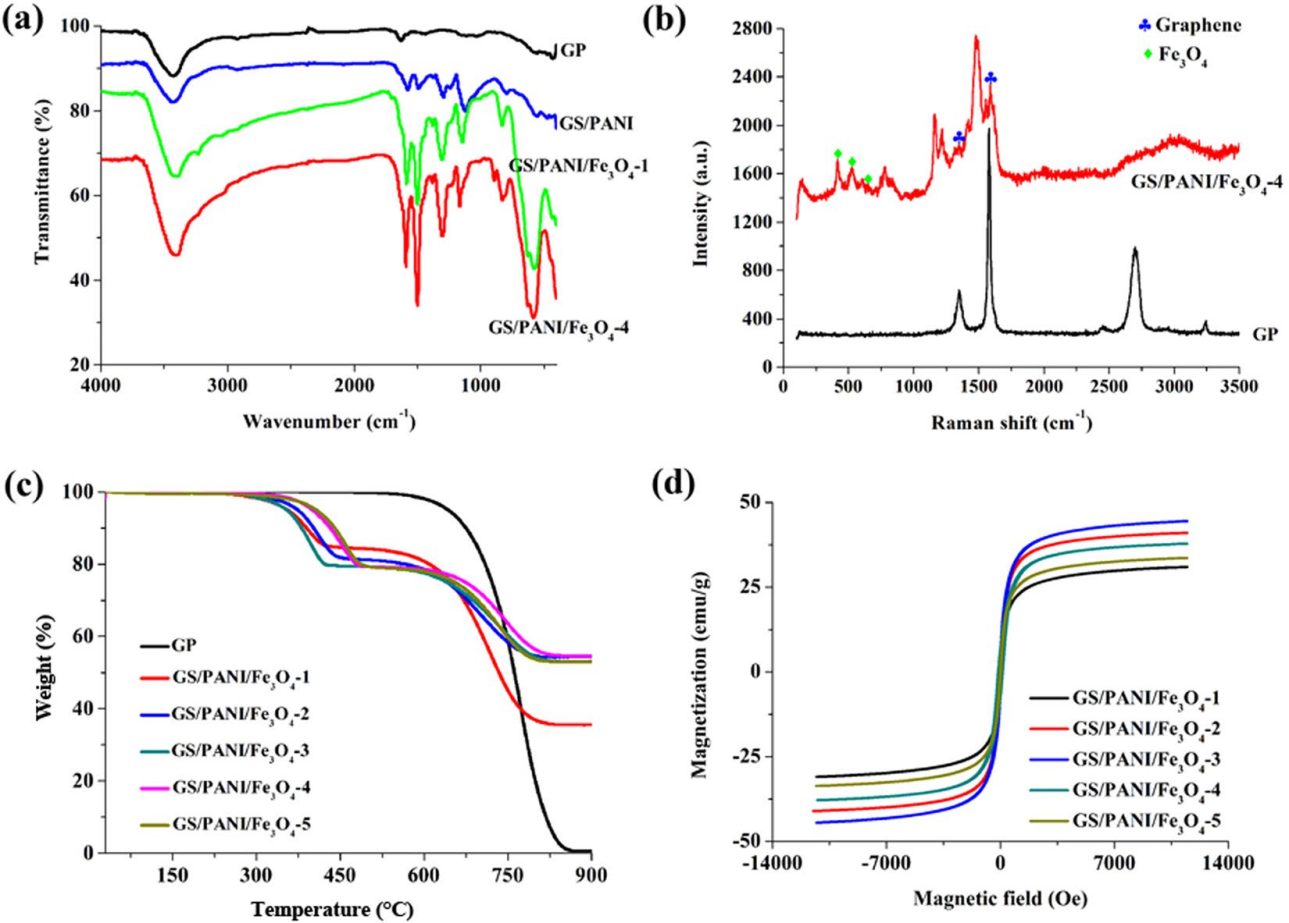
(**a**) FTIR spectra of GP, GS/PANI, GS/PANI/Fe_3_O_4_-1 and GS/PANI/Fe_3_O_4_-4, (**b**) Raman spectra of GP and GS/PANI/Fe_3_O_4_-4, (**c**) TGA curves of GP and GS/PANI/Fe_3_O_4_ nanocomposites, (**d**) The magnetic hysteresis loops of GS/PANI/Fe_3_O_4_ nanocomposites.

FTIR spectra of GP, GS/PANI, GS/PANI/Fe_3_O_4_-1 and GS/PANI/Fe_3_O_4_-4 were analyzed to demonstrate the chemical structure and components of GS/PANI and GS/PANI/Fe_3_O_4_ (Fig. 4a). Compared with the FTIR spectrum of GP, the absorption bands at 1578, 1490, 1245 and 796 cm^−1^ were observed in the spectrum of GS/PANI, which was ascribed to the aromatic C = C stretching vibration of benzenoid ring, the C = N stretching vibration of quinoid ring, C–N^+^ (or C = N^+^) stretching vibration in polar on lattice, and the C-H out of plane bending on 1,2,4-ring, respectively^37, 43^ The absorption band at 1294 cm^−1^ was ascribed to the C–N stretching vibration of secondary aromatic amines owing to the π-electron delocalization induced by protonation of PANI^43^, and the band at 1125 cm^−1^ was assigned to the–NH^+^ = group due to the high level of electron delocalization in Q = NH^+^–B and/or B–NH^+^–B moieties (where Q and B correspond to the quinoid rings and benzenoid rings, respectively) and the hydrogen bonding between –NH^+^– and –N = . For GS/PANI/Fe_3_O_4_, the stretching vibration band of Fe-O was observed at 579 cm^−1^, indicating the presence of Fe_3_O_4_ in the nanocomposites^44^. It was worth noting that the characteristic absorption bands of PANI shifted to higher wavenumber with the introduction of Fe_3_O_4_ (Fig. S2). It might be attributed to the interaction between PANI and Fe_3_O_4_, such as electrostatic interaction and coordination effect^45^. By comparing the FTIR spectra of GS/PANI/Fe_3_O_4_-1 with GS/PANI/Fe_3_O_4_-4, it was observed that the intensity of the characteristic absorption bands of PANI increased with the increase in the molar ratio of aniline to Fe(III).

The structure of the GS/PANI/Fe_3_O_4_ nanocomposites was also studied using Raman spectroscopy. As shown in Fig. 4b, the Raman spectrum of GP showed the characteristic peaks of the *G* band at 1580 cm^−1^ and *G’* band at 2700 cm^−1^ that was the second most prominent peak for graphite samples^46^. The *G* peak was assigned to the doubly degenerate zone center *E*_*2g*_ mode. However, the *G’* band had nothing to do with the *G* peak, but was the second order of zone-boundary phonons. Furthermore, a signal was also detected at 1350 cm^−1^ even in original GP when the analysis was performed at the edge plane despite its intensity was low (*I*_1350_/*I*_G_ = 0.2)^47^. With the introduction of PANI and Fe_3_O_4_, the *G* band of graphene at around 1590 cm^−1^ and its *D* band at around 1345 cm^−1^ were assigned to the in-plane vibration of *sp* ^*2*^ carbon atoms in a 2D hexagonal lattice and the vibration of *sp* ^*3*^ carbon atoms of disordered graphite^28^, respectively. This suggested the original graphite had been well exfoliated due to the intercalation polymerization of aniline and the generation of Fe_3_O_4_ nanoparticles, which was consistent with the XRD results. The new bands at about 420, 525 and 660 cm^−1^ corresponded to the *T*_*2g*_ and *A*_*1g*_ modes of symmetry of Fe_3_O_4_^48^, which confirmed the formation of Fe_3_O_4_ nanoparticles in the nanocomposites. The peaks at 802 and 1160 cm^−1^ were ascribed to C–H out-of-plane bending and C–H in-plane bending in the benzenoid ring, while the bands at 1558 and 1617 cm^−1^ were associated with the C = C stretching vibration in the quinonoid ring and C–C stretching, respectively^49^. In addition, the C = N stretching vibration band of the quinonoid units and the C–N stretching vibration band of the polaronic units appeared at 1489 and 1215 cm^−1^, respectively^50^. Therefore, it can be concluded that graphene, PANI, and Fe_3_O_4_ are present in the nanocomposite.

The thermal stability of GP and GS/PANI/Fe_3_O_4_ nanocomposites were studied by TGA in oxygen atmosphere (Fig. 4c). No weight loss was observed in the temperature range from 30 °C to 260 °C for all samples. The TGA curve of original GP showed almost thorough weight loss due to the decomposition of graphite in oxygen atmosphere. After introducing the Fe_3_O_4_ nanoparticles and PANI, the TGA curves of GS/PANI/Fe_3_O_4_ presented two weight-loss stages. The weight loss between 260~500 °C was attributed to the decomposition of PANI and graphene. The degradation of PANI chains referred to the formation of small aromatic fragments, substituted aromatic fragments, the extended aromatic fragments^51^, and eventually transformed as CO_2_ in oxygen atmosphere. The residuals remaining at 900 °C were Fe_2_O_3_ produced from the oxidation of Fe_3_O_4_ in oxygen atmosphere, and thus the content of Fe_2_O_3_ in GS/PANI/Fe_3_O_4_-1, GS/PANI/Fe_3_O_4_-2, GS/PANI/Fe_3_O_4_-3, GS/PANI/Fe_3_O_4_-4 and GS/PANI/Fe_3_O_4_-5 was calculated to be 35.57%, 52.99%, 54.34%, 54.52% and 53.07%, respectively. According to the content of Fe_2_O_3_ in GS/PANI/Fe_3_O_4_ nanocomposites, the content of Fe_3_O_4_ in GS/PANI/Fe_3_O_4_-1, GS/PANI/Fe_3_O_4_-2, GS/PANI/Fe_3_O_4_-3, GS/PANI/Fe_3_O_4_-4 and GS/PANI/Fe_3_O_4_-5 was 23.71%, 35.33%, 36.23%, 36.35% and 35.38%, respectively.

Figure 4d presents the magnetic hysteresis loops of GS/PANI/Fe_3_O_4_ nanocomposites at 300 K. All samples showed superparamagnetic behavior without magnetic hysteresis and remanence^33^. The saturation magnetization of GS/PANI/Fe_3_O_4_-1, GS/PANI/Fe_3_O_4_-2, GS/PANI/Fe_3_O_4_-3, GS/PANI/Fe_3_O_4_-4 and GS/PANI/Fe_3_O_4_-5 were 30.95, 37.83, 41.02, 44.47 and 33.62 emu•g^−1^, respectively. The saturation magnetization of GS/PANI/Fe_3_O_4_ nanocomposites increased with increasing the molar ratio of aniline to Fe(III), and then decreased as the molar ratio of aniline to Fe(III) to 4:1, which was related to the content of Fe_3_O_4_ in nanocomposites. The higher content of Fe_3_O_4_ corresponded to the greater saturation magnetization. With further increasing the molar ratio of aniline to Fe(III), the content of PANI in the nanocomposites increased, while the content of Fe_3_O_4_ decreased. So, the saturation magnetization decreased with increasing the dosage of aniline. The magnetic property of the GS/PANI/Fe_3_O_4_ nanocomposite made it easily to be separated from aqueous solution by a magnet.

**Figure 5:**
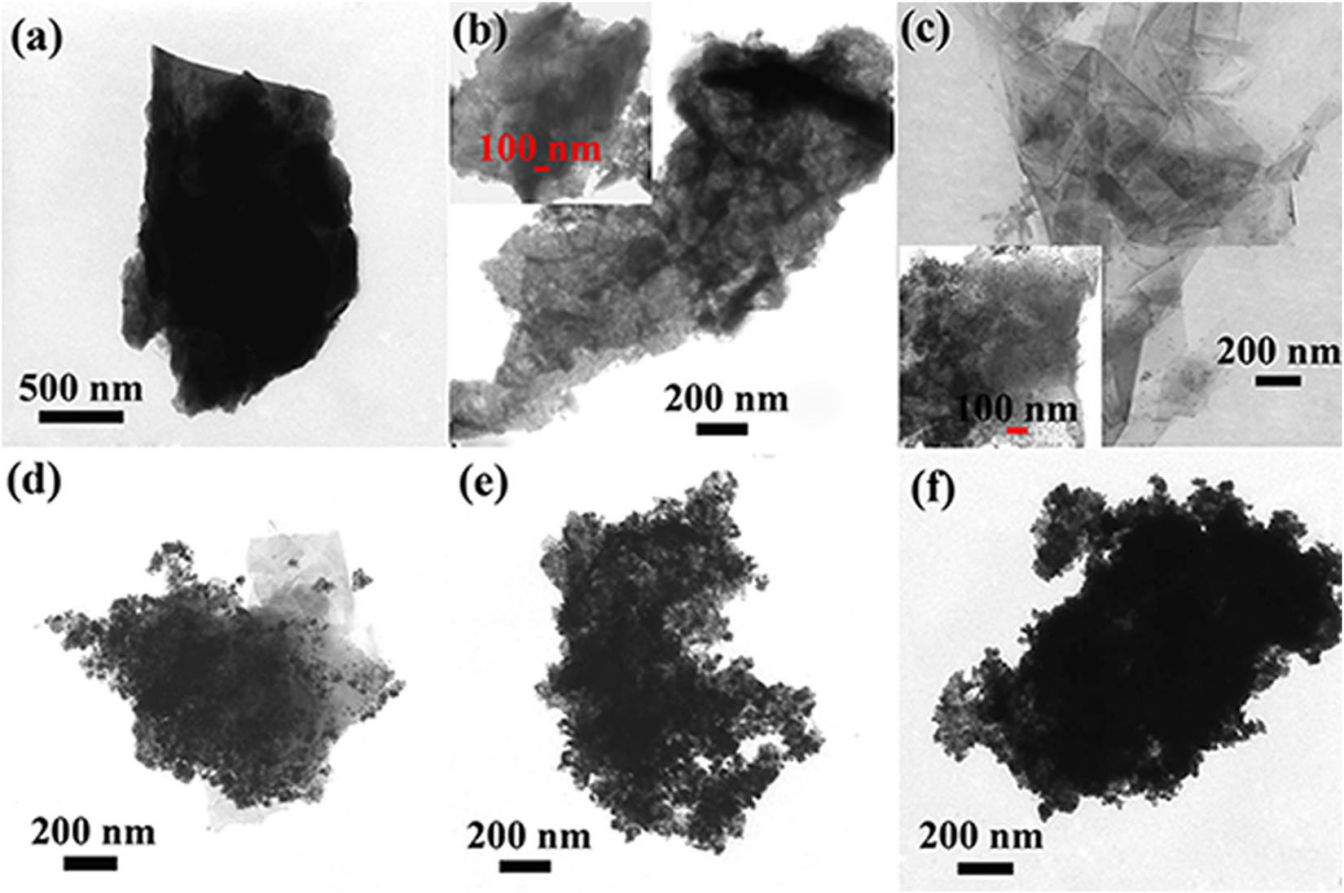
TEM images of (**a**) GP, (**b**) GS/PANI, (**c**) GS/PANI/Fe_3_O_4_-1, (**d**) GS/PANI/Fe_3_O_4_-2, (**e**) GS/PANI/Fe_3_O_4_-3 and (**f**) GS/PANI/Fe_3_O_4_-4.

**Figure 6:**
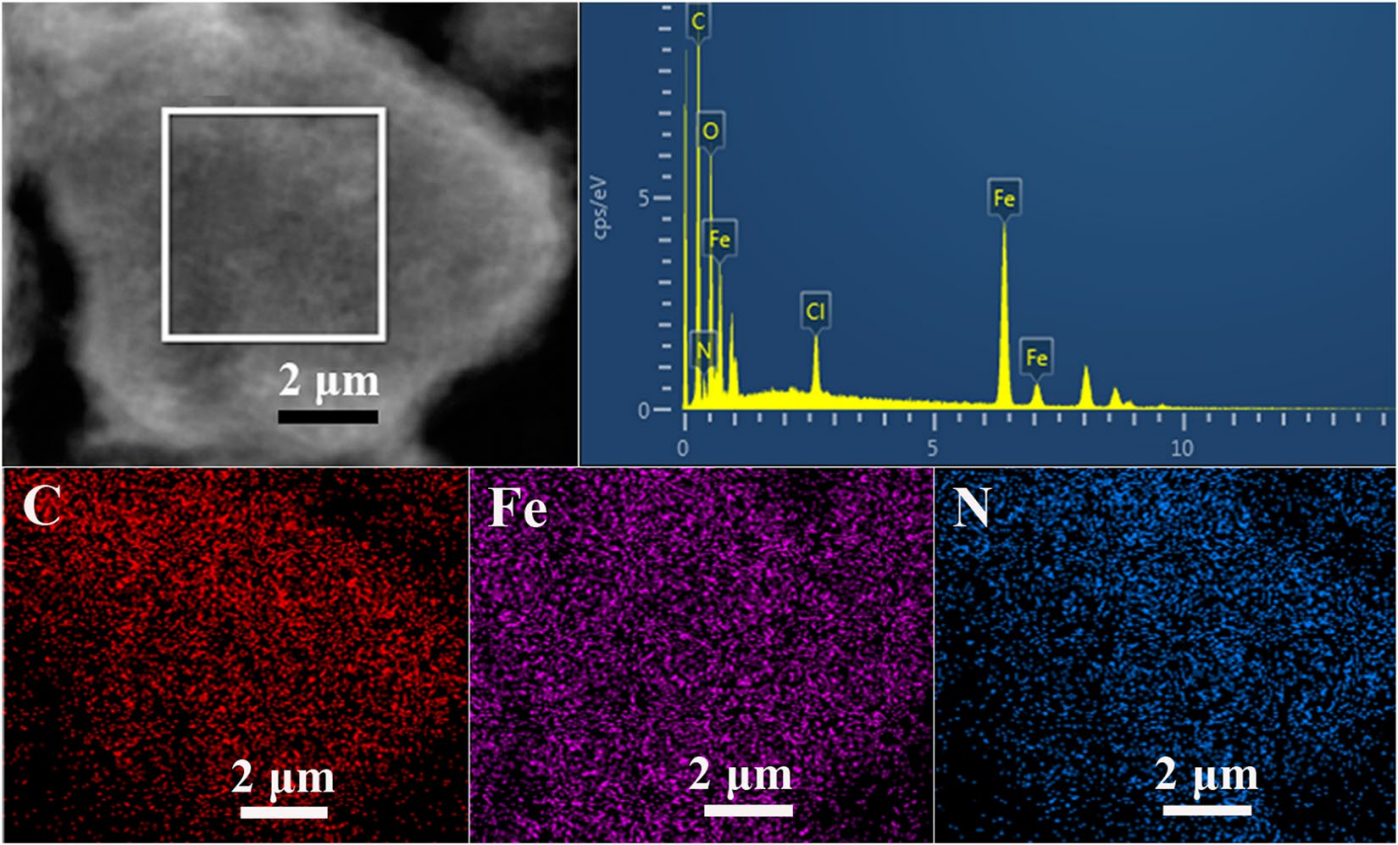
EDS spectrum and elemental mappings of GS/PANI/Fe_3_O_4_-4 nanocomposites.

Figure 5 shows the typical TEM images of GP, GS/PANI, and GS/PANI/Fe_3_O_4_ nanocomposites. It was observed that GP exhibited a flake-like shape (Fig. 5a) with a typical layered structure (Fig. S3a). The key to exfoliate graphite as sheets was to overcome the strong van der Waals-like forces between the layers of graphite *via* physical or chemical methods^52, 53^. Aniline could be protonated and intercalated into the layers of graphite in the solution with the pH lower than 4. The polymerization of the aniline sandwiched in the layers of graphite could destroy the van der Waals forces between the layers and stripped them as thin graphene sheets to obtain the well-defined graphene hybrid structure, as shown in Fig. 5b. Along with the polymerization of aniline, partial Fe(III) was reduced as Fe(II). In the presence of NH_3_∙H_2_O, the Fe_3_O_4_ nanoparticles with an average diameter of about 10 nm were formed on the surface of the graphene sheets by a co-precipitation reaction, which were tightly anchored on the graphene and PANI due to the interaction among them (Fig. 5c). With increasing the molar ratio of aniline to Fe(III), the magnetic nanoparticles anchored on the surface of graphene sheet greatly increased (Fig. 5c~f). In addition, the SEM image, elemental composition and distribution of GS/PANI/Fe_3_O_4_-4 also confirmed that the graphene sheets were completely covered with PANI and Fe_3_O_4_ nanoparticles (Fig. S3b and Fig. 6). It indicated that C, Fe and N elements were evenly distributed on the surface of sheet, and the presence of chlorine element from the EDS spectrum was ascribed to the HCl of the doped PANI, which was in favour of the adsorption of anionic pollutants. Furthermore, it also revealed that graphene sheets could effectively induce the uniform encapsulation of PANI and Fe_3_O_4_ nanoparticles on the surface of graphene without the free aggregations of PANI and Fe_3_O_4_ nanoparticles.

### Adsorption of dye and magnetic recycling

**Figure 7:**
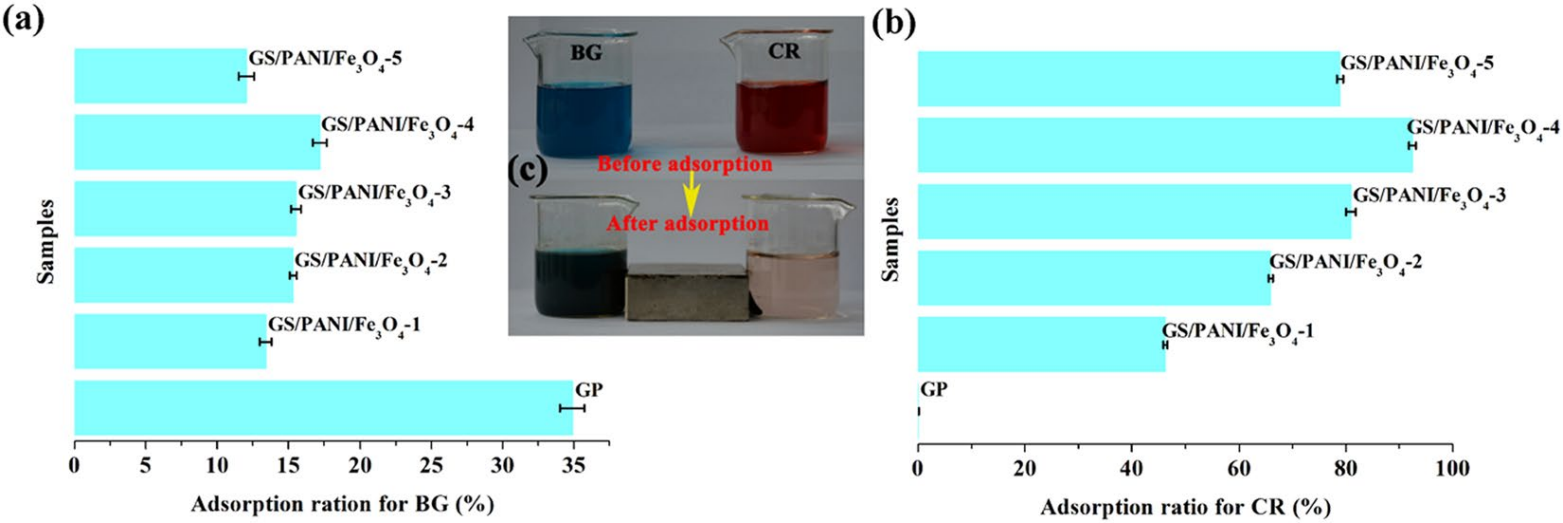
Adsorption ratio of the nanocomposites toward different dye solutions (**a**) 100 ppm of BG, (**b**) 100 ppm of CR. (**c**) Digital photographs of 100 mg/L of BG and CR solution before and after adsorption using GS/PANI/Fe_3_O_4_-4 as adsorbent.

**Figure 8:**
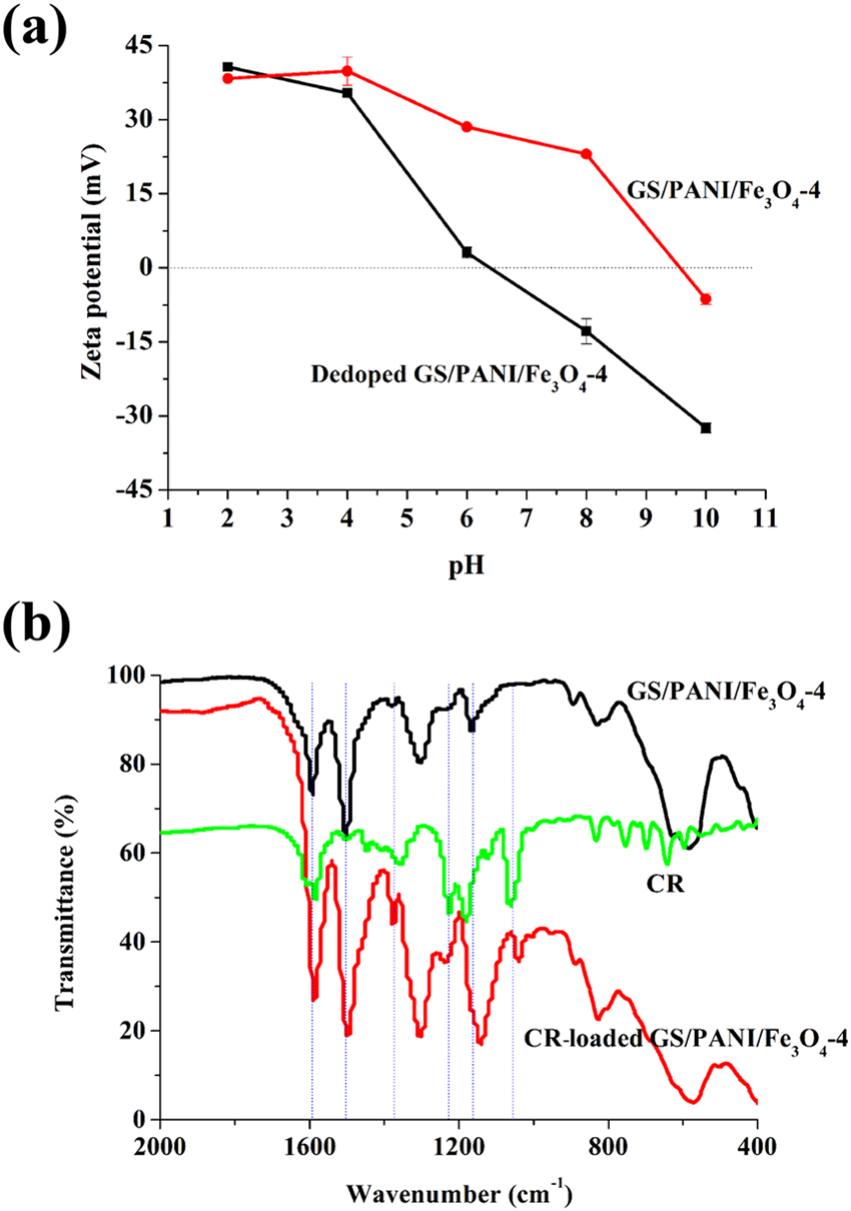
(**a**) Zeta potential of GS/PANI/Fe_3_O_4_-4 and the dedoped GS/PANI/Fe_3_O_4_-4 suspension (1 mg mL^−1^) at different pH values (The measurement was repeated three times under the same conditions). (**b**) FTIR spectra of GS/PANI/Fe_3_O_4_-4, CR and CR-loaded GS/PANI/Fe_3_O_4_-4.

The GS/PANI/Fe_3_O_4_ nanocomposites are potential to be used for water treatment, such as the removal of dye molecules due to their special superparamagnetism and excellent adsorption performance. As shown in Fig. 7a, GS/PANI/Fe_3_O_4_ nanocomposite exhibited poor adsorption efficiency to the basic dye BG compared with that of GP (34.9%) in the 100 mg/L of aqueous solution. This phenomenon might be attributed to the electrostatic repulsion between BG molecules and GS/PANI/Fe_3_O_4_ nanocomposites. The surface electrical characteristic of GS/PANI/Fe_3_O_4_-4 and the dedoped GS/PANI/Fe_3_O_4_-4 at different pH values is depicted in Fig. 8a. It could be seen that the isoelectric point (pH of zero point charge, pHpzc) of GS/PANI/Fe_3_O_4_-4 was about 9.6. It suggested that the GS/PANI/Fe_3_O_4_-4 nanocomposite was positively charged at pH < pHpzc, but negatively charged at pH > pHpzc due to the different ionization degree of functional groups at different pH values. Compared with the dedoped GS/PANI/Fe_3_O_4_-4, the isoelectric point of GS/PANI/Fe_3_O_4_-4 shifted toward higher pH value, which was mainly ascribed to the protonation of amino groups of PANI. Therefore, GS/PANI/Fe_3_O_4_ composite exhibited a poor adsorption ratio to the cationic BG (pH = 6.3) while it presented a high adsorption ratio toward anionic CR. Due to the same principle, GP almost showed no adsorption to CR (0.02%) (Fig. 7b). Among all of GS/PANI/Fe_3_O_4_ nanocomposites, the adsorption ratio of GS/PANI/Fe_3_O_4_-4 to CR reached 92.4% due to the presence of more active adsorption sites in it. Furthermore, GS/PANI/Fe_3_O_4_-4 could also be rapidly separated from solution using a magnet (Fig. 7c), indicating that GS/PANI/Fe_3_O_4_ nanocomposites were easily to be recycled by an external magnetic field. In order to investigate the adsorption mechanism, FTIR spectra of GS/PANI/Fe_3_O_4_-4 before and after adsorption of CR were analyzed in the region of 400~2000 cm^−1^ (Fig. 8b). The characteristic absorption bands of PANI at 1590 cm^−1^, 1500 cm^−1^ and 1130 cm^−1^ shifted to low wavenumber region after adsorption of CR due to the decrease in the electron density of benzene ring of polyaniline. This might be attributed to the electrostatic interaction, hydrogen bond and the *π-π* stacking interaction between PANI and CR^54^.

**Figure 9:**
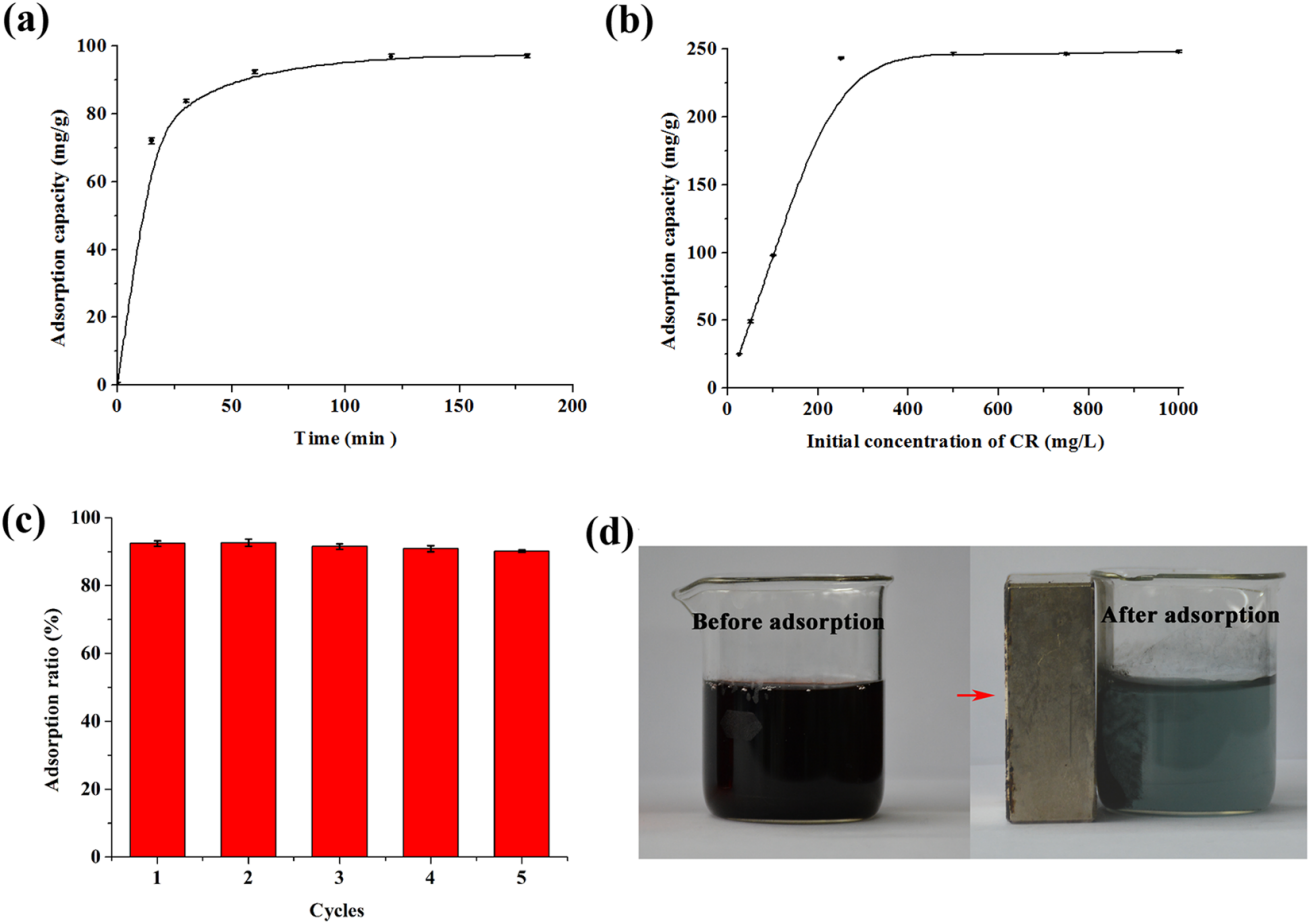
(**a**) Effect of contact time on adsorption of GS/PANI/Fe_3_O_4_-4 to CR, (**b**) Effect of initial concentration of CR on the adsorption of GS/PANI/Fe_3_O_4_-4 to CR, (**c**) Adsorption ratio for 100 mg/L CR as a function of adsorption-desorption cycle, and (**d**) Digital photographs of the mixed solution composed of 50 mg/L of BG and CR before and after adsorption using GS/PANI/Fe_3_O_4_-4.

Figure 9a presents the effects of contact time on the adsorption of GS/PANI/Fe_3_O_4_-4 to CR. The adsorption equilibrium was reached within 2 h. In order to study the adsorption kinetics of GS/PANI/Fe_3_O_4_-4 to CR, the pseudo first-order (Eq. 2) and pseudo second-order (Eq. 3) kinetic models were used to fit the adsorption data:^55^

2 $$\mathrm{ln}({q}_{e}-{q}_{t})=\,\mathrm{ln}\,{q}_{e}-{k}_{1}\times t$$

3 $$t/{q}_{t}=1/({k}_{2}\times {{q}_{e}}^{2})+t/{q}_{e}$$

where *q*_*e*_ and *q*_*t*_ are the adsorption capacities of the adsorbents for CR (mg/g) at equilibrium state and time *t* (min), respectively. *k*_1_ (min^−1^) is the pseudo first-order rate constant, *k*_2_ (g (mg·min)^−1^) is the pseudo second-order kinetic rate constant. The above relevant parameters could be calculated from the slope and intercept of the ln(*q*_*e*_–*q*_*t*_) vs *t* and *t*/*q*_*t*_ vs *t* plots, as listed in Table S1. The theoretical *q*_*e*_ obtained from pseudo-second-order model was more close to the experimental values than that calculated from the pseudo first-order model. As shown in Figs S4 and S5, the fitting curves from pseudo second-order model exhibited higher linear correlation coefficients (R^2^) than that obtained from pseudo first-order kinetic model. This suggested that the pseudo second-order kinetic model was more suitable to express the adsorption behaviors of GS/PANI/Fe_3_O_4_-4 to CR. Therefore, the adsorption process of CR on GS/PANI/Fe_3_O_4_-4 was probably controlled by the chemical adsorption process^56^.

In order to achieve a saturated adsorption capacity for further comparison with the reported adsorbents, the effect of initial concentrations of CR on the adsorption capacity of GS/PANI/Fe_3_O_4_-4 ranging from 25 to 1000 mg/L was studied, as shown in Fig. 9b. It was observed that the adsorption capacities of GS/PANI/Fe_3_O_4_-4 to CR increased with the increase in the initial CR concentration until it reached the adsorption saturation at the initial concentration of 500 mg/L. At lower concentration, sufficient vacant active sites were available, and the limited adsorbates couldn’t fully occupy the adsorption sites of the adsorbent. Therefore, GS/PANI/Fe_3_O_4_-4 to CR exhibited a good adsorption ratio as the initial concentration of CR was lower than 250 mg/L. With the continuous increase in the initial concentration of CR, the available adsorption sites were almost completely occupied by dye molecules, and then the adsorption capacities reached the maximum and almost kept the equilibrium state. In order to describe the adsorption process and the possible mechanism, the Langmuir (Eq. 4) and Freundlich (Eq. 5) isotherm models were employed to analyze the experimental data:^57, 58^

4 $${C}_{e}/{q}_{e}=1/({q}_{m}\times b)+{C}_{e}/{q}_{m}$$

5 $$\mathrm{log}\,{q}_{{\rm{e}}}=\,\mathrm{log}\,{K}_{{\rm{F}}}+1/n\times \,\mathrm{log}\,{C}_{{\rm{e}}}$$

where *q*_*e*_ (mg/g) is the adsorption capacities of the adsorbents for CR at equilibrium, *C*_e_ is the concentration of CR solution after adsorption (mg/L), *q*_*m*_ is the maximum adsorption capacity (mg/g), *b* is the Langmuir adsorption constant (L/mg), which is related to the free energy of adsorption. *K*_F_ ((mg/g)(L/mg)^1/n^) is Freundlich constant and roughly defined as the adsorption capacity, and the constant 1/n represents the adsorption intensity. As shown in Table S2, the linear correlation coefficient (*R* ^2^ = 0.9999) obtained from the results fitting with Langmuir isotherm model (Fig. S6) was higher than that obtained from Freundlich isotherm model (*R* ^2^ = 0.5460) (Fig. S7). In addition, the experimental adsorption capacity of 248.76 mg/g was almost equal to the theoretical value of 248.12 mg/g calculated from Langmuir isotherm model. This suggested that the Langmuir isotherm model was well fitted with the adsorption data, and only monolayer adsorption, instead of immigration of adsorbate, occurred on the surface^57^. Table S3 lists the maximum adsorption capacities of the reported graphene-based adsorbents and the as-prepared GS/PANI/Fe_3_O_4_ nanocomposite toward CR. It was clear that GS/PANI/Fe_3_O_4_ nanocomposite exhibited high adsorption capacity to CR. Especially, GS/PANI/Fe_3_O_4_ nanocomposite was fabricated by a one-pot method that the exfoliation of graphite and the functionalization of graphene could be simultaneously achieved, with no need to prepare GO by a complex and high-cost procedure. Therefore, it is feasible to be used for the large-scale industrial fabrication of highly efficient graphene-based adsorbents.

Figure 9c shows that GS/PANI/Fe_3_O_4_-4 can be easily recycled by magnetic separation and reused for 5 cycles after regenerated using 0.5 M NaOH solution as the desorbing agent. No obvious decrease of adsorption ratio was observed after 5 adsorption-desorption cycles, and the slight decrease might be attributed to the incomplete desorption of CR from adsorbents. In addition, the GS/PANI/Fe_3_O_4_ nanocomposites could be employed to remove the mixed dyes of cationic BG and anionic CR. Interestingly, GS/PANI/Fe_3_O_4_ nanocomposite exhibited good adsorption ratio toward the mixed dyes due to its little affinity to cationic dye BG. GS/PANI/Fe_3_O_4_-4 nanocomposite could simultaneously remove BG and CR of the mixed solution, with the adsorption ratio of 82.7% and 96.5% within 1 h for BG and CR, respectively (Fig. 9d). This phenomenon might be associated to the electrostatic interaction and hydrogen bond between BG and CR, which could be confirmed by the UV-vis spectra of the mixed dye solution before and after adsorption with GS/PANI/Fe_3_O_4_-4 (Fig. S8). It is well-known that CR is a typical anionic dye with amido and sulfo groups while BG is a cationic dye with diethylamino groups, as illustrated in Fig. S9. So the UV-vis spectrum of the mixed solution composed of CR and BG was different from the single CR solution and BG solution due to the interaction between CR and BG. After addition of adsorbents, anionic CR was preferential adsorbed on the GS/PANI/Fe_3_O_4_ nanocomposite due to the electrostatic interaction, hydrogen bond and the *π-π* stacking interaction between PANI and CR, which was confirmed by the FTIR spectrum of GS/PANI/Fe_3_O_4_-4 after adsorption of CR (Fig. 7b). And then the cationic BG was adsorbed on adsorbents *via* the electrostatic between the BG and the CR loaded on GS/PANI/Fe_3_O_4_-4.

## Conclusions

In summary, we have successfully fabricated the superparamagnetic GS/PANI/Fe_3_O_4_ nanocomposites *via* a simple, green and industrially feasible approach. The exfoliation and functionalization of graphite was achieved under mild conditions *via* one-pot method based on the redox reaction between aniline and Fe(III). The obtained nanocomposites can be easily separated by magnetic field, and exhibit a good adsorption ratio of 92.4% toward 100 mg/L of CR within 2 h. The adsorption kinetics and the adsorption isotherm are well fitted by pseudo second-order kinetic model and Langmuir isotherm model, respectively, and the maximum adsorption capacity is about 248.76 mg/g. In addition, GS/PANI/Fe_3_O_4_ nanocomposites also can be employed to remove the mixed dyes composed of cationic BG and anionic CR, involving the preferential adsorption of the anionic dye CR, and then the cationic BG. It is worth noting that this method might be more beneficial to large-scale production of graphene-based adsorbents for environment applications.

## Materials and Methods

### Materials

Native graphite powder (GP, <40 mm) was purchased from Qingdao Huatai Lubricant Sealling S&T Co. Ltd, Qingdao, China. BG was purchased from Sinopharm Chemical Reagent Co., Ltd, Shanghai, China. CR was purchased from Alfa Aesar Co. Ltd. FeCl_3_∙6H_2_O, aniline, and other reagents with analytical reagent grade were purchased from Tianjin Chemical Co., Tianjin, China, and used without further purification. Ultrapure water (18.25 MΩ cm) was used throughout the experiment.

### Preparation of GS/PANI/Fe_3_O_4_ nanocomposites

In a typical procedure, 0.5 g of GP was added into 50 mL of water containing aniline, and the pH value was adjusted to 4 using 1.0 M HCl solution. The mixture was ultrasonically treated for 1 h to make sure that the protonated aniline was intercalated into the interlayer of graphite. And then FeCl_3_∙6H_2_O was added into above solution and the reaction was stirred for 8 h at room temperature. Then, the reaction system was heated to 70 °C, and 10 mL of NH_3_∙H_2_O (14 wt %) was added dropwise to the above mixture under vigorous stirring for 1 h to form the magnetic nanoparticles. Before being dried under vacuum, the obtained products were treated with 0.1 M HCl solution, and then rinsed with water for several times. In order to investigate the effect of the molar ratio of aniline/Fe(III) on the structure of GS/PANI/Fe_3_O_4_ nanocomposites and their adsorption property to dyes, the GS/PANI/Fe_3_O_4_ nanocomposites with different content of PANI and magnetic nanoparticles were also prepared, and the preparation conditions of the samples were summarized in Table 1.

**Table 1:** The conditions of the samples preparation.

Samples	FeCl_3_·6H_2_O/g	Graphite/g	Aniline/mL	NH_3_·H_2_O/mL	n_anili_ _ne_: n _Fe(III)_
GS/PANI/Fe_3_O_4_-1	3.552	0.5	0.6	10	1:2
GS/PANI/Fe_3_O_4_-2	3.552	0.5	1.2	10	1:1
GS/PANI/Fe_3_O_4_-3	3.552	0.5	2.4	10	2:1
GS/PANI/Fe_3_O_4_-4	3.552	0.5	3.6	10	3:1
GS/PANI/Fe_3_O_4_-5	3.552	0.5	4.8	10	4:1
GS/PANI	3.552	0.5	3.6	0	3:1

As a control, the products without magnetic nanoparticles (GS/PANI) were also prepared under the same procedure without the addition of NH_3_∙H_2_O, and the preparation conditions were also presented in Table 1.

### Dye adsorption experiments

The adsorption of GS/PANI/Fe_3_O_4_ nanocomposites toward BG and CR was performed in a series of conical flasks containing 25 mg of the adsorbents and 25 mL 100 ppm of BG or CR, respectively. The mixtures were shaken in a thermostatic shaker at 25 °C for 1 h, and then GS/PANI/Fe_3_O_4_ nanocomposites were separated by a magnet. The dye concentration in the solution was analyzed using ultraviolet and visible (UV-vis) spectrophotometer by monitoring the adsorption behavior at a wavelength of maximum absorbance (625 nm and 498 nm for BG and CR, respectively). The adsorption ratio of GS/PANI/Fe_3_O_4_ nanocomposites toward dyes was calculated from the dye concentrations in solutions before and after adsorption according to Eq. 6:

6 $$Adsorptionratio=({C}_{0}\,-{C}_{e})/{C}_{0}\times 100{\rm{ \% }}$$

where *C*_0_ and *C*_e_ are the concentration of dye solutions before and after adsorption, respectively.

The influence of the contact time and the initial concentration was investigated by evaluating the adsorption of GS/PANI/Fe_3_O_4_-4 to CR. In addition, GS/PANI/Fe_3_O_4_ nanocomposite was used to evaluate the adsorption to the mixed dye solution composed of 50 mg/L of CR and 50 mg/L of BG. The desorption and regeneration studies were performed as follows: the adsorbent GS/PANI/Fe_3_O_4_-4 was separated after adsorption of CR and immersed into 25 mL of NaOH solution (0.5 M) for 4 h. Then, the adsorbents were washed with distilled water for several times and regenerated with 0.5 M HCl solution for 2 h. Finally, the adsorbents were washed with distilled water for several times and used for next adsorption process. The adsorption ratio toward CR was calculated from the dye concentrations in solutions before and after adsorption. All adsorption experiments were undertaken in triplicate under the identical conditions and mean values were reported in this paper.

## Characterizations

Bruker IFS 66 v/s IR spectrometer (Bruker, Karlsruhe, Germany) was used for the Fourier transform infrared spectroscopy analysis in the range of 400~4000 cm^−1^ with the resolution of 4 cm^−1^. The morphologies of the samples were observed using a JEM-1200 EX/S transmission electron microscope (TEM) (JEOL, Tokyo, Japan). The samples were dispersed in ethanol and vibrated for 10 min and then deposited on a copper grid covered with a perforated carbon film. The surface morphology of samples was observed using scanning electron microscope (SEM, JSM-6701F, JEOL, Ltd. Japan). The samples were fixed on the copper stubs and then coated with gold for the SEM observation. The elemental composition and distribution were determined using a Kevex energy dispersive spectrometer (EDS). Thermogravimetric analysis (TGA) was performed on a Perkin Elmer STA6000 thermogravimetric analyzer under oxygen atmosphere at a heating rate of 10 °C min^−1^. The X-ray diffraction (XRD) analysis was conducted with an X-ray powder diffractometer with Cu anode (PAN analytical Co. X’pert PRO), running at 40 kV and 30 mA. Magnetic properties of samples were detected by vibrating sample magnetometer (Lakeshore 7304). The X-ray photoelectron spectroscopy (XPS) analysis was carried out on a PHI 5,702 spectrometer equipped with a monochromatic Al Ka X-ray source. UV-vis absorption spectra were measured at room temperature by using Lambda 35 UV-vis spectrometer (PerkinElmer, U.S.A.). The zeta potential of GS/PANI/Fe_3_O_4_-4 in deionized water was determined with a Malvern Zetasizer Nano system with irradiation from a 633 nm He-Ne laser (ZEN3600). As a control, the zeta potential of the dedoped GS/PANI/Fe_3_O_4_-4 was also analyzed after being dedoped using NaOH.

## Electronic supplementary material

Supplementary Information
