# Supplementary material for: Facile fabrication of superparamagnetic graphene/polyaniline/Fe3O4 nanocomposites for fast magnetic separation and efficient removal of dye
Source: Sci Rep. 2017 Jul 13;7:5347. doi: 10.1038/s41598-017-05755-6 (PMC5509721; doi:10.1038/s41598-017-05755-6)
Supplement: Supplementary file 1 — Supplementary Information [file 41598_2017_5755_MOESM1_ESM.pdf]

Facile fabrication of superparamagnetic graphene/polyaniline/Fe_3O_4 nanocomposites for fast magnetic separation and efficient removal of dye

Bin Mu[1], Jie Tang[1,2], Long Zhang[3], and Aiqin Wang[1]

[1]State Key Laboratory of Solid Lubrication, Center of Eco-materials and Green Chemistry, Lanzhou Institute of Chemical Physics, Chinese Academy of Sciences, Lanzhou 730000, China.

[2] University of Chinese Academy of Sciences, Beijing 100049, PR China.

[3] School of Material Science and Engineering, Lanzhou University of Technology, Lanzhou 730050, China.

*Corresponding author, Tel.: +86 931 4968118; fax: +869318277088. Email: aqwang@licp.cas.cn (A.Q. Wang)

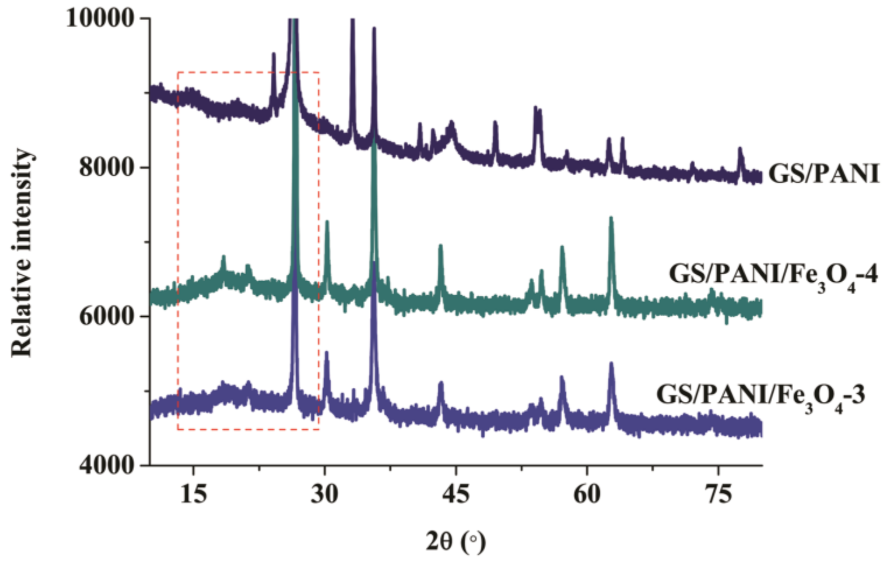

Fig. S1 Partially enlarged view of XRD patterns of GS/PANI, GS/PANI/Fe_3O_4-3, and GS/PANI/Fe_3O_4-4.

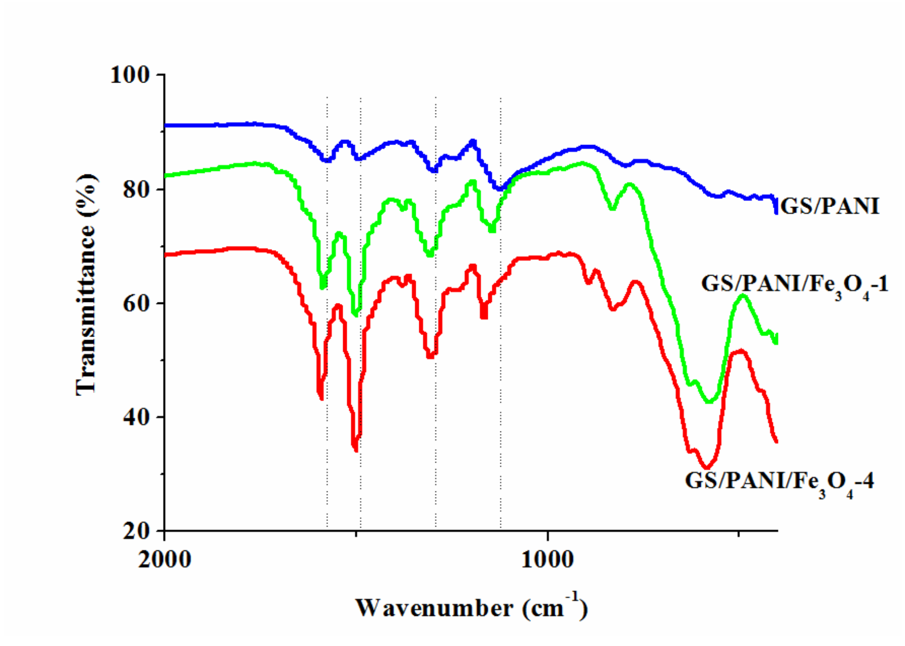

Fig. S2 Partially enlarged view of FTIR spectra of GS/PANI, GS/PANI/Fe_3O_4-1, and GS/PANI/Fe_3O_4-4.

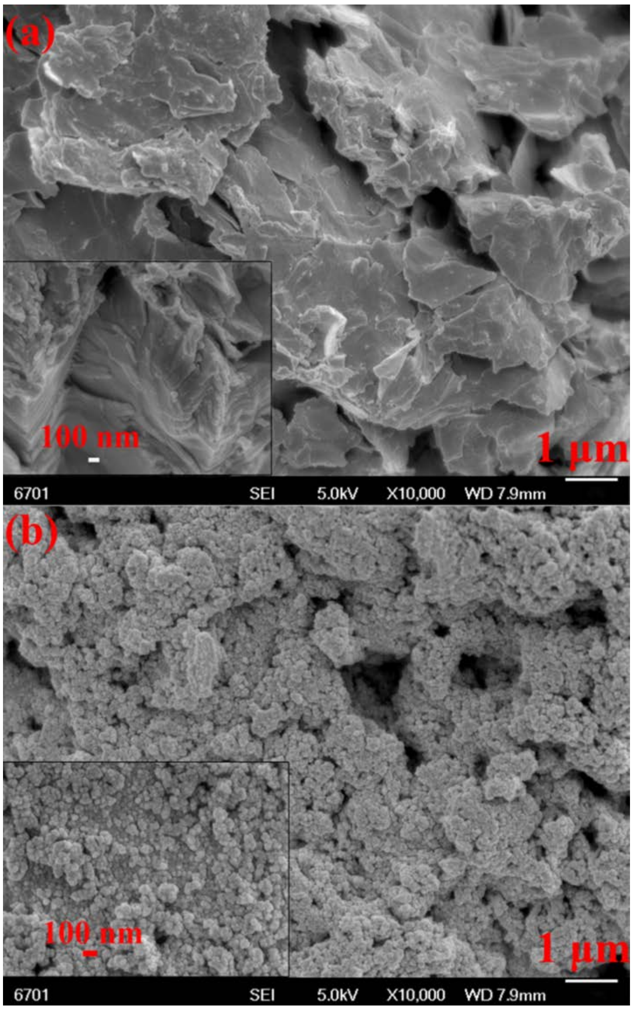

Fig. S3 SEM images of (a) GP and (b) GS/PANI/Fe_3O_4-4.

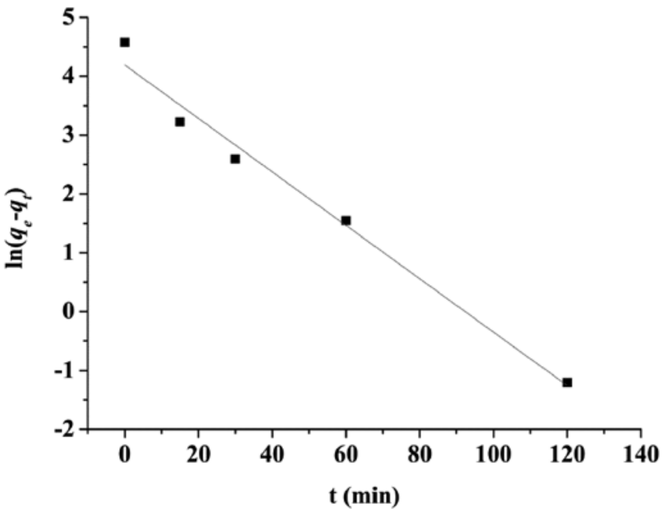

Fig. S4 The fitting plots of ln(q_e-q_t) versus *t* by pseudo-first-order kinetic model for the adsorption of CR (100 mg/L).

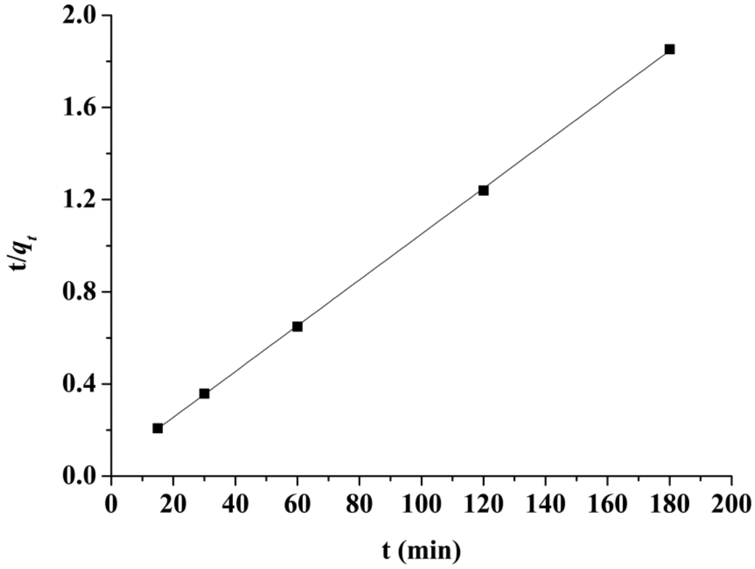

Fig. S5 The fitting plots of t/q_t versus *t* by pseudo-second-order kinetic model for the adsorption of CR (100 mg/L).

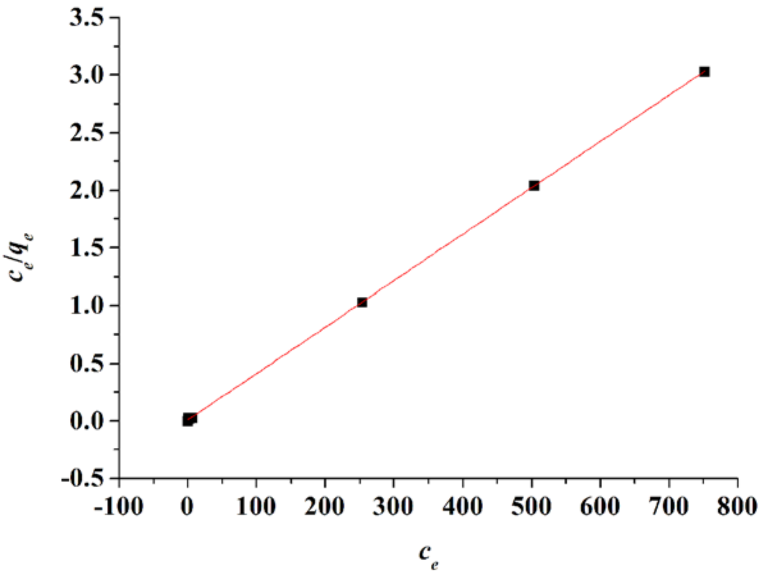

Fig. S6 The fitting plots of c_e/q_e versus c_e with Langmuir isotherm model for the adsorption of CR.

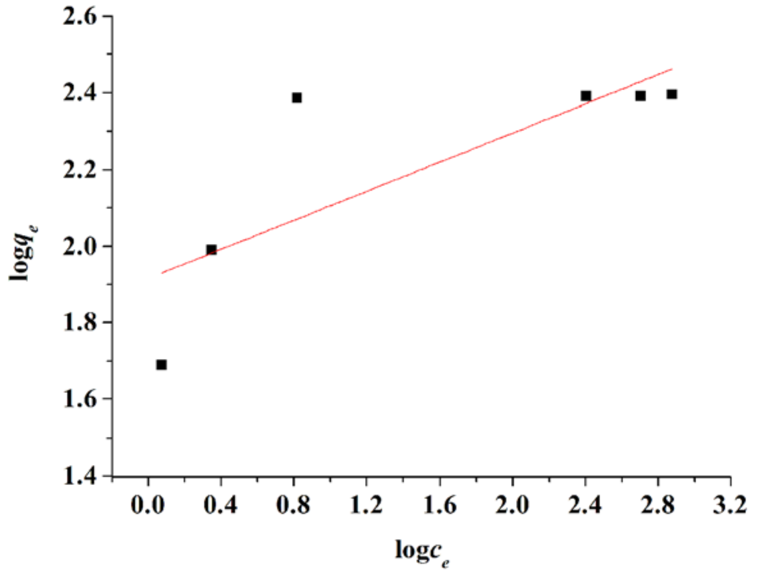

Fig. S7 The fitting plots of $\log q_e$ versus $\log c_e$ with Freundlich isotherm model for the adsorption of CR.

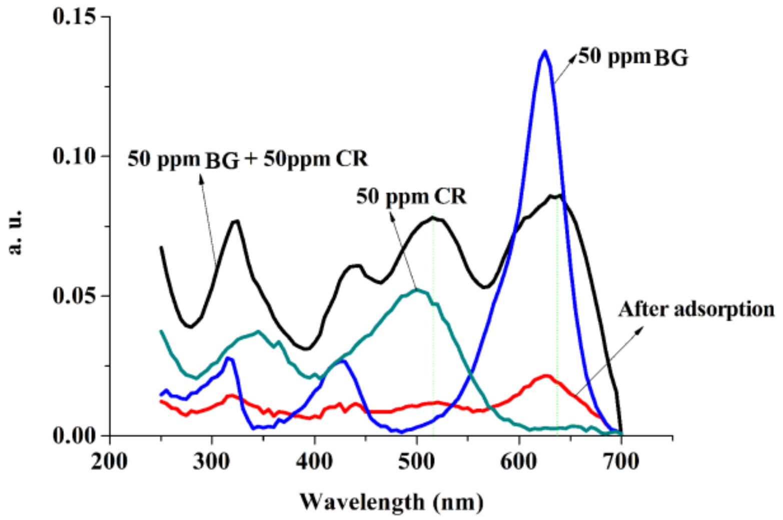

Fig. S8 UV-vis spectra of 50 ppm of BG, 50 ppm of CR, and the mixed solution composed of 50 ppm of BG and CR before and after adsorption.

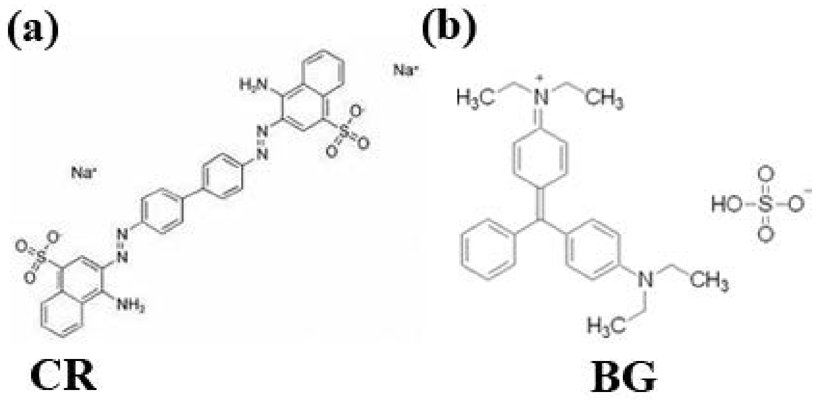

Fig. S9 Structural formula of (a) CR and (b) BG.

Table S1 Adsorption kinetic parameters of GS/PANI/Fe_3O_4-4 to CR.

Sample	q_{exp} (mg/g)	Pseudo first-order model			Pseudo second-order model		
		q_e (mg/g)	k_1 (min^{-1})	R^2	q_e (mg/g)	k_2 (min^{-1})	R^2
GS/PANI/Fe_3O_4-4	97.12	65.98	0.0454	0.9790	100.50	0.0018	0.9999

Table S2 Isotherm parameters for the adsorption of CR onto GS/PANI/Fe_3O_4-4.

Sample	q_{exp} (mg/g)	Langmuir model			Freundlich model		
		q_m (mg/g)	b (L/mg)	R^2	n	K_F	R^2
GS/PANI/Fe_3O_4-4	248.12	248.76	0.4674	0.9999	5.2715	82.41	0.5460

Table S3 Comparison of the maximum adsorption capacity of the reported graphene-based adsorbents toward CR with the as-prepared GS/PANI/Fe_3O_4 nanocomposites

Samples	Precursor	q_{max} (mg/g)	References
NiO/graphene nanosheets	GO	123.89	S1
pTSA-PANI@graphene-PVC	graphene	45.45	S2
Doped PVC@graphene-PANI	graphene	26.31~40.0	S3
Graphene oxide/chitosancomposite fibers	GO	175.4	S4
Magnetic mesoporous titanium dioxide–graphene oxide	GO	89.95	S5
Ce-Fe/graphene hybrids	GO	179.50	S6
Magnetic Fe_3O_4@graphene nanocomposites	GO	33.66	S7
Graphene oxide/chitosan fibers	GO	294.12	S8
Fe_3O_4/graphene oxide nanocomposites	GO	98.8	S9
GS/PANI/Fe_3O_4 nanocomposites	Natural graphite powder	248.76	This study

References

S1. Rong, X. S. *et al.* A facile hydrothermal synthesis, adsorption kinetics and isotherms to Congo Red azo-dye from aqueous solution of NiO/graphene nanosheets adsorbent. *J. Ind. Eng. Chem.* **26**, 354-363 (2015).

S2. Ansari, M. O. *et al*. Facile strategy for the synthesis of non-covalently bonded and para-toluene sulfonic acidfunctionalized fibrous polyaniline@graphene-PVC nanocomposite for the removal of Congo red. *New J. Chem.* **39**, 7004-7011 (2015).

S3. Kumar, R., Ansari, M. O., Parveen, N., Barakat M. A. & Cho M. H. Simple route for the generation of differently functionalized PVC@graphene-polyaniline fiber bundles for the removal of Congo red from wastewater. *RSC Adv.* **5**, 61486-61494 (2015).

S4. Li, Y. H. *et al*. Mechanical and dye adsorption properties of graphene oxide/chitosancomposite fibers prepared by wet spinning. *Carbohyd. Polym.* **102**, 755-761 (2014).

S5. Li, L. L. *et al*. Removal of Congo Red by magnetic mesoporous titanium dioxide-graphene oxide core-shell microspheres for water purification. *Dalton Trans.* **43**, 8431–8438 (2014).

S6. Ling, Q., Yang, M., Li, C. S. & Zhang, A. M. Preparation of highly dispersed Ce-Fe bimetallic oxides on graphene and their superior adsorption ability for Congo red. *RSC Adv.* **4**, 4020-4027 (2014).

S7. Yao Y. J. *et al*. Synthesis, characterization, and adsorption properties of magnetic Fe_3O_4@graphene nanocomposite. *Chem. Eng. J.* **184**, 326-332 (2012).

S8. Du, Q. J. *et al*. Highly enhanced adsorption of congo red onto graphene oxide/chitosan fibers by wet-chemical etching off silica nanoparticles. *Chem. Eng. J.* **245**, 99-106 (2014).

S9. Namvari, M. & Namazi, H. Clicking graphene oxide and Fe_3O_4 nanoparticles together: an efficient adsorbent to remove dyes from aqueous solutions. *Int. J. Environ. Sci. Technol.* **11**, 1527-1536 (2014)
